# Single Atrium Repair with Delayed Sternal Closure in a Postpartum Multiparous Woman: A Case Report

**DOI:** 10.3390/jcm15176732

**Published:** 2026-08-30

**Authors:** Jianing Wang, Huimin Shao, Can Xu, Dongjin Wang

**Affiliations:** 1 Department of Thoracic and Cardiovascular Surgery, Nanjing Drum Tower Hospital, Chinese Academy of Medical Sciences & Peking Union Medical College, Nanjing 210008, China; 2 Department of Thoracic and Cardiovascular Surgery, Nanjing Drum Tower Hospital, The Affiliated Hospital of Nanjing University Medical School, Nanjing 210008, China; 3 Institute of Cardiothoracic Vascular Disease, Nanjing University, Nanjing 210008, China; 4 Department of Ultrasound Medicine, Nanjing Drum Tower Hospital, The Affiliated Hospital of Nanjing University Medical School, Nanjing 210008, China

**Keywords:** single atrium, surgical correction, thoracic aorta suture, pregnancy, case report

## Abstract

**Background/Objectives:** Single atrium is a rare congenital heart defect typically repaired in infancy. Unrepaired adults with pregnancy face high risk, and postpartum repair with delayed sternal closure (DSC) has rarely been reported. **Methods:** We present a 33-year-old multiparous woman who presented at 30 weeks + 1 day of gestation with progressive dyspnea. Transthoracic echocardiography (TTE) revealed a single atrium with a common atrioventricular valve and an estimated pulmonary artery systolic pressure (PASP) of 47 mmHg. The multidisciplinary team advised early cesarean section, which was performed at 30 weeks + 3 days of gestation, but postpartum symptoms persisted. Six months later, the patient underwent surgical correction. The procedure was converted from right subaxillary mini-thoracotomy to median sternotomy due to intraoperative aortic dissection, which was repaired. The single atrium was partitioned using a bovine pericardial patch. Due to myocardial edema and hemodynamic instability, the sternum was left open and closed on postoperative day 4. She required 8 days of mechanical ventilation and 13 days of intensive care, and was discharged on day 27. **Results:** Surgical repair was successful, and at the 6-month postoperative follow-up, she had achieved NYHA class I functional status, with an estimated PASP of 40 mmHg and a well-positioned baffle with no residual shunt. **Conclusions:** This case demonstrates that multidisciplinary collaboration is essential for pregnant patients with unrepaired congenital heart disease, postpartum surgical correction is feasible with careful perioperative planning, and DSC is a safe strategy for managing hemodynamic instability in complex cardiac cases.

## 1. Introduction

Single atrium is a rare congenital heart defect characterized by the complete absence of the interatrial septum, accounting for less than 1% of all congenital heart diseases [1]. In this anomaly, oxygenated and deoxygenated blood mix within a common atrial chamber, leading to variable degrees of cyanosis, volume overload, and ultimately pulmonary hypertension (PH) if left uncorrected [2]. Most patients undergo surgical correction in infancy or early childhood [1]. However, a subset of patients, particularly in resource-limited settings or those with mild symptoms, may reach adulthood without surgical intervention [2,3].

Pregnancy in women with unrepaired single atrium poses substantial maternal and fetal risks. The physiological changes of pregnancy, including a 30–50% increase in blood volume and cardiac output, place significant hemodynamic stress on patients with intracardiac shunts [4]. In a common atrial chamber with bidirectional shunting, long-standing volume overload may lead to progressive pulmonary vascular remodeling and eventually PH, a well-recognized risk factor for adverse pregnancy outcomes [5]. The rarity of this condition is underscored by a Chinese retrospective study identifying single atrium in only 0.6% of pregnant women with PH [6]. In the context of repeated pregnancies, this risk is amplified by the cumulative hemodynamic burden [7,8], as illustrated by our patient, who completed three pregnancies before surgical repair, highlighting that even in patients who tolerate an initial pregnancy, subsequent gestations may accelerate clinical deterioration and warrant heightened surveillance.

To date, only a limited number of case reports have described pregnancy outcomes in women with single atrium. Kojqiqi et al. reported a 27-year-old woman with single atrium who experienced three preterm births and one miscarriage before diagnosis and subsequent successful surgical repair using a double-velour patch [1]. A case of successfully managed pregnancy in a patient with complex cyanotic congenital heart disease has also been reported [9]. Garg et al. reported a 25-year-old female with single atrium who presented with shortness of breath and a history of recurrent abortions [10].

Despite these reports, the optimal management strategy for women with unrepaired single atrium who present during pregnancy, including the timing of delivery, perioperative planning, and postpartum surgical correction, remains poorly defined. Moreover, the use of delayed sternal closure (DSC) in the context of postpartum single atrium repair has not been previously described. We report a case of single atrium diagnosed during the third pregnancy in a 33-year-old woman who underwent successful surgical repair 6 months postpartum. DSC was employed due to intraoperative aortic dissection and severe myocardial edema, highlighting the importance of surgical flexibility in high-risk congenital heart disease.

This case report has been prepared in accordance with the CARE (CAse REport) guideline.

## 2. Case Report

A 33-year-old multiparous woman (gravida 3, para 2), with a height of 1.55 m, weight of 67.5 kg, and body mass index (BMI) of 28.1 kg/m^2^, presented at 30 weeks and 1 day of gestation with progressive dyspnea on exertion and palpitations. Her obstetric history was notable for one prior uncomplicated vaginal delivery and one cesarean section, with a resulting uterine scar. Her comorbidities included gestational diabetes mellitus, uterine myomas, and retinal atrophy. The retinal atrophy, identified in childhood, was accompanied by night blindness and poor daytime vision. She had no history of tuberculosis, hepatitis, or trauma. Additional surgical history included surgical interventions for polydactyly. There was no prior diagnosis of congenital heart disease.

On admission, the patient’s SpO_2_ was 85–88% on room air and improved to 88–92% with supplemental oxygen (5 L/min via nasal cannula). Arterial blood gas analysis on admission (on 5 L/min oxygen) showed a pH of 7.44, PaCO_2_ at 34 mmHg, PaO_2_ at 65 mmHg, SpO_2_ at 94%, hemoglobin at 15.4 g/dL, and lactate at 1.3 mmol/L. These findings indicated only mild hypoxemia, without evidence of secondary erythrocytosis or severe cyanosis.

Transthoracic echocardiography (TTE) performed during the antenatal evaluation unexpectedly revealed a single atrium with a common atrioventricular valve and an estimated pulmonary artery systolic pressure (PASP) of 47 mmHg. The right ventricle was dilated, with a basal diameter of 44 mm, and right ventricular systolic function was moderately reduced. Left ventricular ejection fraction (LVEF) was 61.8%. A multidisciplinary consultation involving the Department of Cardiology, Cardiac Surgery, and Critical Care Medicine was convened. Given the TTE findings of a single atrium with bidirectional shunting, oxygen saturation levels of 85–88% on room air, and the gestational age of 30 weeks and 1 day, the multidisciplinary team recommended early pregnancy termination followed by surgical evaluation for definitive correction.

At 30 weeks + 3 days of gestation, an elective cesarean section was performed, with delivery of a healthy neonate. The postpartum course was uneventful, and the patient was discharged with continued medical therapy.

**Figure 1:**
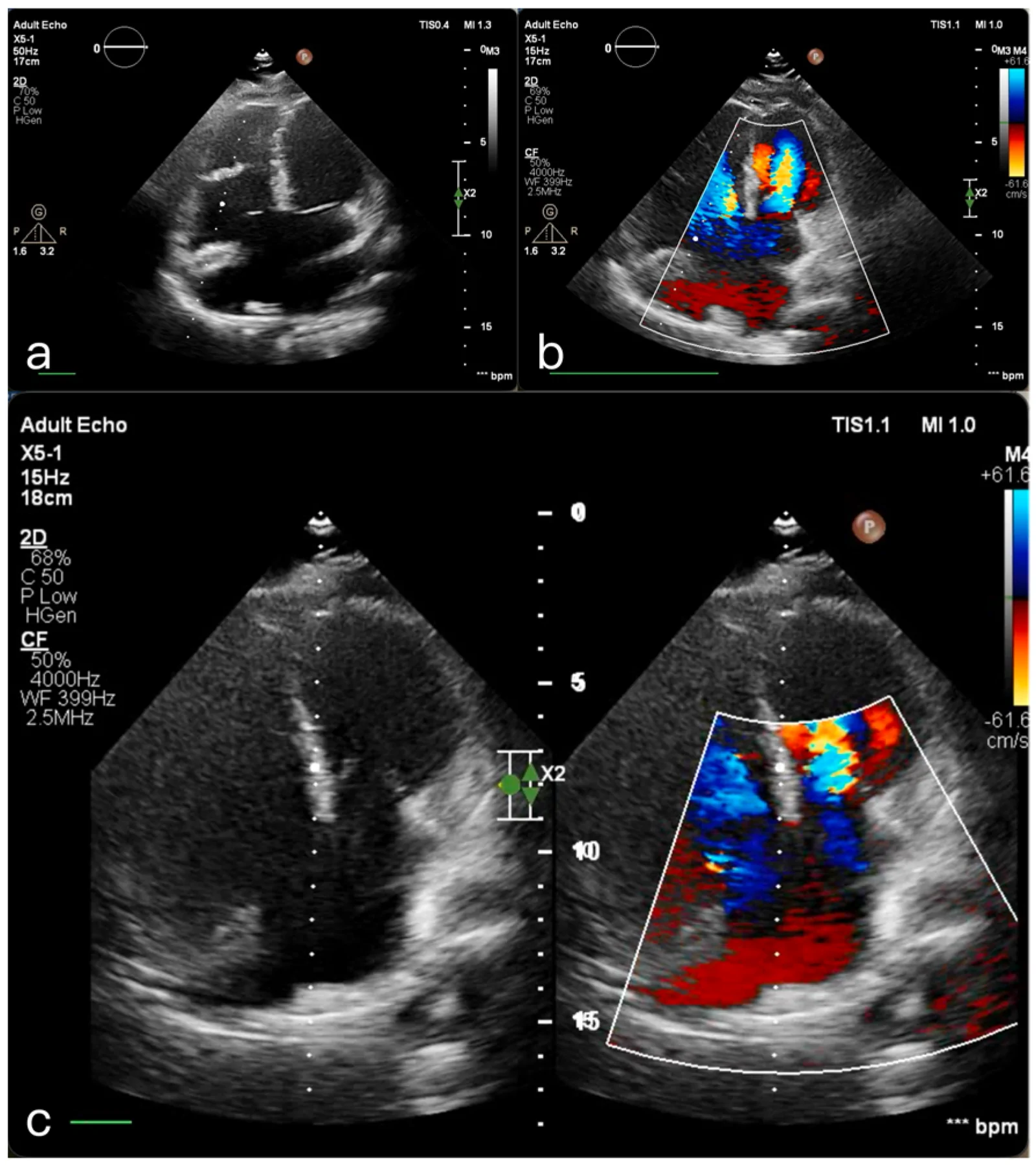
Preoperative transthoracic echocardiography (TTE) (apical four-chamber view) reveals a single atrium. (**a**) Two-dimensional imaging shows complete absence of the interatrial septum, with a common atrial chamber. (**b**) Color Doppler demonstrates bidirectional shunting across the common atrium. (**c**) Side-by-side comparison of two-dimensional and color Doppler imaging at the same plane highlights the anatomical defect and its corresponding hemodynamic disturbance in the common atrial chamber.

The patient was readmitted 6 months postpartum for definitive surgical evaluation. Preoperative workup demonstrated persistent right ventricular enlargement, with a basal diameter of 48 mm at 6 months postpartum compared with 44 mm during pregnancy, and elevated estimated PASP of 38 mmHg. LVEF was 62.1%. Preoperative computed tomography angiography (CTA) was suggestive of single atrium, which was consistent with the echocardiographic diagnosis of single atrium (Figure 1), and further revealed left lung ventilation–perfusion mismatch, as suggested by heterogeneous ventilation with patchy mosaic attenuation changes (Supplementary Figure S1). This finding was interpreted as a chronic secondary change due to long-standing hemodynamic alterations in the setting of single atrium, and did not affect surgical planning.

Preoperative CTA confirmed that all four pulmonary veins drained directly into the common atrium, with no anomalous pulmonary venous connection. Aortic sinus was approximately 25 mm, ascending aorta was approximately 32 mm, aortic arch was approximately 22 mm, and proximal descending aorta was approximately 18 mm. All segments demonstrated smooth vessel walls with no luminal narrowing, dilation, tortuosity, dissection flap, or coarctation. No features suggestive of connective tissue disease were observed (Supplementary Figure S2). Therefore, no surgical intervention was required on the pulmonary veins, and the procedure was limited to creation of a neo-interatrial septum using a bovine pericardial patch.

After thorough risk–benefit assessment, the patient was deemed a suitable candidate for surgical correction of the single atrium. At 6 months postpartum, surgery was undertaken due to persistent symptoms and right ventricular enlargement despite partial resolution of pregnancy-related hemodynamic stress. Informed consent was obtained after a detailed discussion of surgical risks, including bleeding, arrhythmias, low cardiac output syndrome, and the potential need for DSC, as well as other potential perioperative complications.

**Figure 2:**
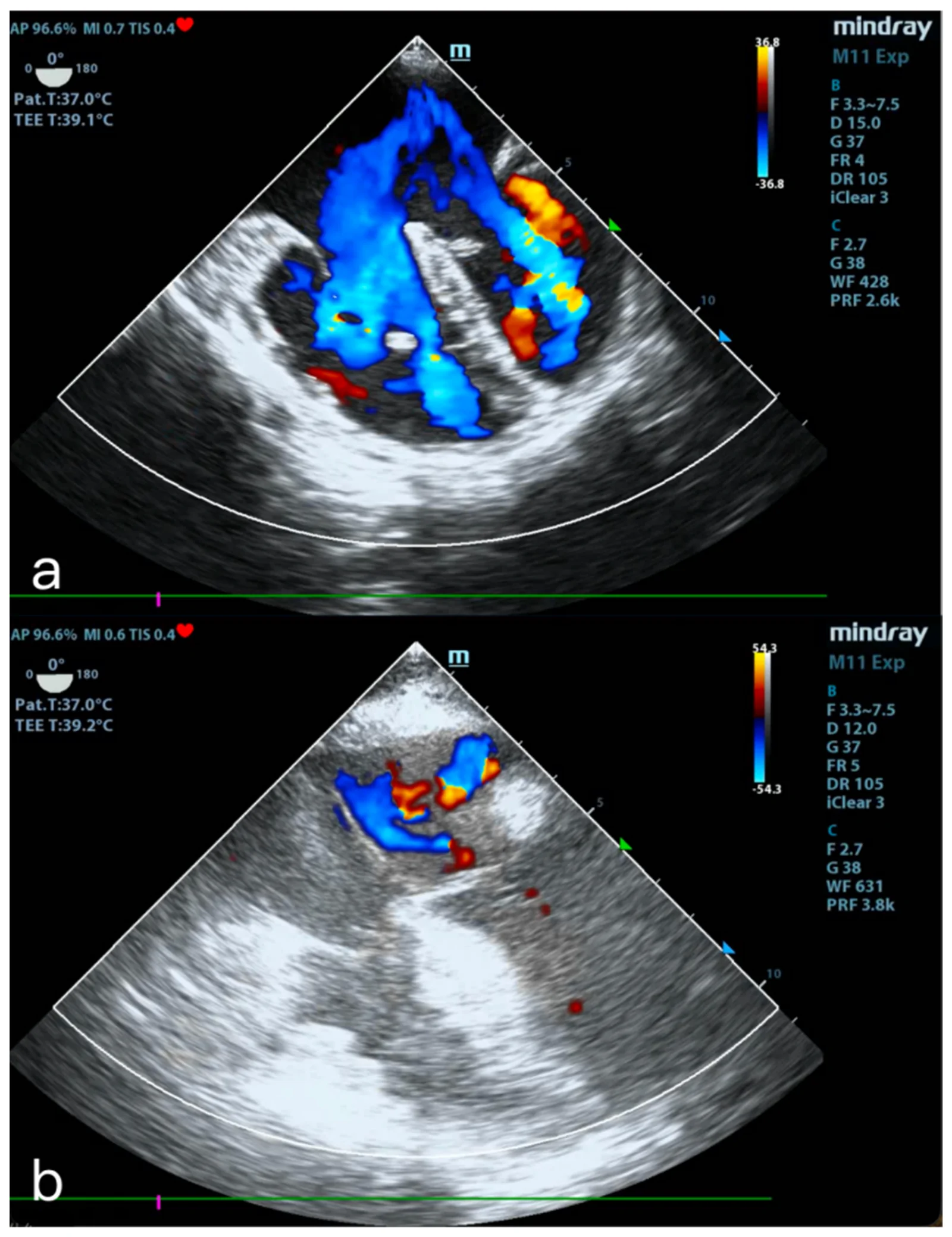
Intraoperative transesophageal echocardiography (TEE) (mid-esophageal four-chamber view) before and after surgical correction. (**a**) The pre-repair image shows a common atrial chamber with complete absence of the interatrial septum and reveals disturbed flow within the common atrium. (**b**) Post-repair image demonstrates the neo-interatrial septum created with a bovine pericardial patch; color Doppler confirms no residual shunt across the neo-septum.

Surgery was performed under general anesthesia with cardiopulmonary bypass (CPB) via a right subaxillary mini-thoracotomy approach. CPB was established through ascending aortic and bicaval cannulation. After aortic cross-clamping and cardioplegic arrest, the right atrium was opened. Intraoperative inspection revealed a single atrium with no interatrial septum; the pulmonary veins drained normally into the common atrium. Surgical correction was performed using a bovine pericardial patch to create a new interatrial septum (Figure 2). The patch was tailored and sutured to partition the common atrium into separate left and right atria, directing pulmonary venous return to the left-sided chamber and systemic venous return to the right-sided chamber, with careful attention to preserve atrioventricular valve function and avoid obstruction of the superior and inferior vena cavae. The coronary sinus was routed to the right atrium.

Detailed TTE and intraoperative assessment demonstrated a common atrial chamber with a common atrioventricular valve (the anterior leaflet of the mitral valve and the septal leaflet of the tricuspid valve were observed at the same attachment level in the apical four-chamber view, with no leaflet number anomalies; mild-to-moderate regurgitation of both valves), intact ventricular septum, normal pulmonary venous drainage (all four pulmonary veins drained directly into the common atrium, with no anomalous connection), and normal systemic venous drainage (the caval veins drained directly into the common atrium), confirming the diagnosis of single atrium with a common atrioventricular valve.

**Figure 3:**
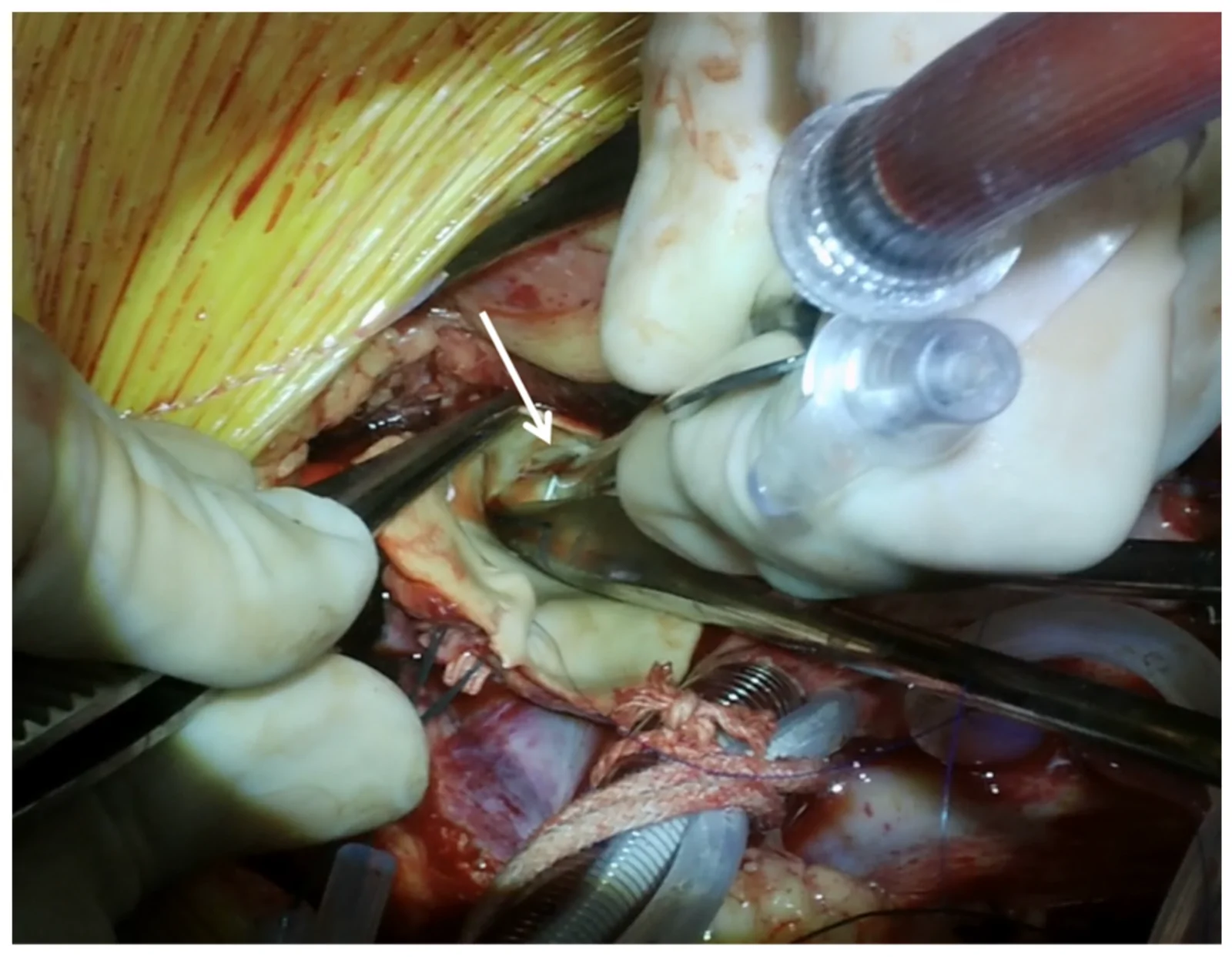
Intraoperative photograph showing an aortic dissection (white arrow) encountered during surgical correction of single atrium.

Intraoperatively, elevated central venous pressure (approximately 40 mmHg) was observed, and the heart was noted to be shifted rightward into the right thoracic cavity. Aortic dissection was suspected. Intraoperative transesophageal echocardiography (TEE) demonstrated findings suggestive of aortic dissection. After consultation with the patient’s family, the procedure was converted to a median sternotomy. Intraoperative exploration confirmed a Stanford type B (DeBakey type IIIa) aortic dissection, with an entry tear located approximately 1 cm distal to the origin of the left subclavian artery (Figure 3). No other aortic arch branch vessels were involved, and no end-organ malperfusion was identified. The dissection tear was repaired with 5-0 Prolene continuous suture.

Following rewarming and weaning from CPB, the patient exhibited hemodynamic instability. Due to significant myocardial edema and multiple unsuccessful attempts at sternal closure resulting in circulatory compromise, the decision was made to leave the sternum open with delayed closure. A sterile occlusive dressing was applied, and the patient was transferred to the intensive care unit with the sternum temporarily open. Total CPB time was 655 min and aortic cross-clamp time was 236 min. DSC was successfully performed on postoperative day 4.

The patient’s early postoperative course was critical. She required 8 days of mechanical ventilation and 13 days in the intensive care unit (ICU). No major complications, including low cardiac output syndrome, malignant arrhythmias, mediastinal infection, or cerebrovascular events, were observed. The chest tubes, pericardial drains, mediastinal drains, and subcutaneous drains were gradually removed. She was transferred to the general ward on postoperative day 13 and discharged home on postoperative day 27 with stable hemodynamic status. At discharge, she was prescribed routine postoperative medications.

**Figure 4:**
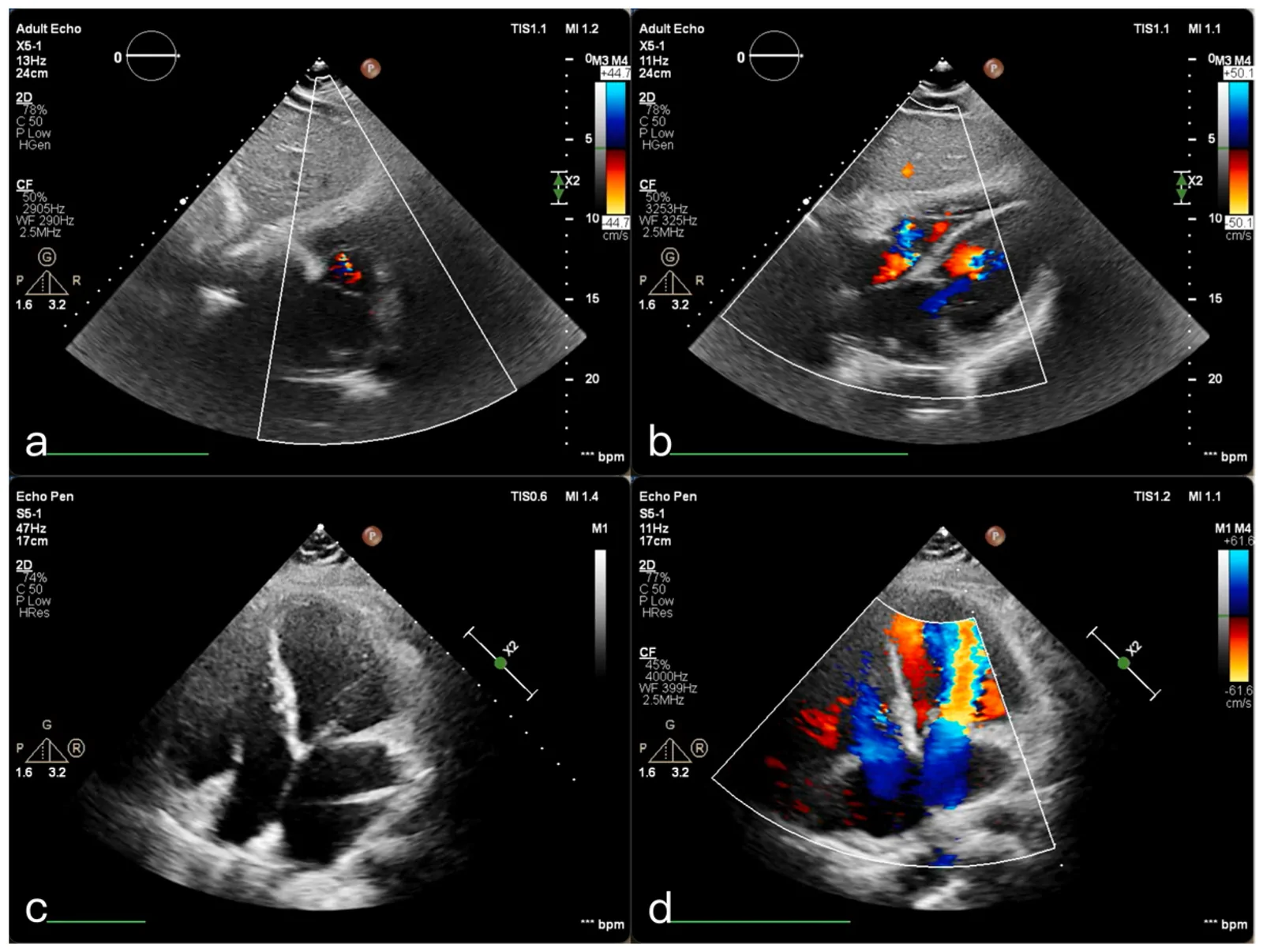
Postoperative follow-up TTE: (**a**) Subcostal two-chamber view at 3 months confirms the integrity of the bovine pericardial baffle. (**b**) Subcostal four-chamber view at 3 months postoperatively demonstrates the neo-interatrial septum in a good position, with no residual shunt on color Doppler. (**c**) Apical four-chamber view at 6 months shows a stable neo-septum with preserved cardiac chamber geometry. (**d**) Color Doppler at 6 months confirms no residual shunt across the baffle.

At 3 months postoperatively, the patient reported significant symptomatic improvement, with NYHA class II functional status and no limitations in daily activities. TTE demonstrated a well-positioned bovine pericardial baffle with no residual shunt; elevated PASP was 43 mmHg; right ventricular basal diameter was 40.5 mm; LVEF was 50.5%. At 6 months postoperatively, her functional improvement was sustained, with NYHA class I. TTE confirmed stable baffle position, no residual shunt, and no valvular regurgitation. Elevated PASP was 40 mmHg, right ventricular basal diameter remained at 40.5 mm, and LVEF was 50.2%. The patient has been scheduled for annual TTE surveillance (Figure 4). No adverse events were observed throughout the 6-month follow-up.

The patient demonstrated good adherence to the prescribed postoperative medication regimen. Adherence was assessed through clinical interview and medication reconciliation at each follow-up visit. The patient tolerated the medications well, with no reported adverse effects. Regular TTE surveillance confirmed sustained hemodynamic improvement, further supporting therapeutic compliance. The patient’s clinical course from symptom onset through postoperative follow-up is summarized in Table 1.

## 3. Discussion

Single atrium is an uncommon congenital cardiac anomaly, with a reported prevalence of less than 1% among all congenital heart defects. The condition is typically diagnosed and surgically corrected in early childhood [1]. However, our patient remained undiagnosed until 33 years of age, when she presented with progressive dyspnea during her third pregnancy. In summary, this case contributes unique insights into the late presentation of unrepaired single atrium, the multidisciplinary management of pregnancy complicated by right-sided overload, and the successful surgical rescue of an unexpected aortic dissection with DSC.

First, our case underscores the substantial maternal risks associated with pregnancy in women with an unrepaired single atrium. Pregnancy induces a 30–50% increase in blood volume and cardiac output, which imposes a significant hemodynamic burden on patients with pre-existing intracardiac shunts [4]. Pregnancy in women with PH is associated with substantial maternal morbidity and mortality, with risk particularly elevated in those with severe pulmonary vascular disease and Eisenmenger physiology [7,8,11]. One prospective cohort study reported a mortality rate of 6.6% in pregnant women with PH [11], while other recent series have reported rates ranging from 11% to 25% [7]. Mortality remains substantially higher in Eisenmenger syndrome, with reported rates of 15% in a contemporary cohort and up to 26% in a literature review [8,11]. The 2022 ESC/ERS Guidelines for the diagnosis and treatment of PH emphasize that pregnancy in women with pulmonary arterial hypertension (PAH) and other forms of severe PH should be managed in experienced PH centers with multidisciplinary teams, and that women with intermediate- or high-risk profiles should be advised against pregnancy [7].

Literature on pregnancy in women with unrepaired single atrium remains limited to isolated case reports, most of which describe unfavorable maternal and fetal outcomes, including preterm delivery, miscarriage, and thromboembolic complications [1,9,10]. In our patient, TTE performed for progressive dyspnea at 30 weeks and 1 day of gestation revealed a single atrium. A multidisciplinary evaluation was promptly convened, and early pregnancy termination was recommended to mitigate the risks of right-sided volume overload and to create a safe window for subsequent surgical correction. This experience reinforces that a multidisciplinary heart team approach, involving cardiologists, obstetricians, anesthesiologists, and cardiac surgeons, is essential for optimizing maternal and fetal outcomes in such high-risk pregnancies.

Second, the optimal timing of surgical correction in postpartum patients with unrepaired congenital heart disease remains a clinical challenge. While some patients may experience symptomatic improvement after delivery as the hemodynamic burden of pregnancy resolves, in this patient, definitive repair was undertaken 6 months postpartum after partial resolution of pregnancy-related hemodynamic stress, but with persistent symptoms, elevated estimated PASP, and persistent right ventricular enlargement on serial imaging, with a basal diameter of 48 mm at 6 months postpartum compared with 44 mm during pregnancy. Right ventricular basal diameter decreased from 48 mm preoperatively to 40.5 mm at 3 months postoperatively and remained stable at 40.5 mm at 6 months. Functional status improved from NYHA class III preoperatively to class II at 3 months and class I at 6 months postoperatively. Surgical repair undertaken at 6 months postpartum was associated with favorable functional and hemodynamic outcomes at 3 and 6 months follow-up, though longer-term surveillance is warranted to monitor right ventricular function and PASP trajectory.

Third, our case illustrates the importance of intraoperative decision-making and flexibility in surgical strategy. Preoperative CTA excluded any pre-existing aortic dissection or wall abnormality. Aortic cannulation and CPB initiation were performed without incident. The dissection was first identified during the single atrium repair procedure, when central venous pressure rose progressively and the heart shifted rightward. This sequence and the absence of pre-existing aortic pathology on preoperative imaging raise the possibility of an iatrogenic mechanism. This chronology underscores a critical point: even in the absence of pre-existing aortic disease, iatrogenic dissection can occur during complex congenital heart surgery. Prompt recognition, decisive conversion to sternotomy, direct repair, and judicious use of DSC were essential to achieving a favorable outcome. To the best of our knowledge, the intraoperative occurrence of aortic dissection requiring surgical repair during single atrium correction has not been previously reported, making this case particularly instructive for surgeons managing complex congenital heart disease in adults.

Fourth, the use of a bovine pericardial patch for atrial septation in our patient yielded excellent early results. At 6 months postoperatively, echocardiography confirmed a well-positioned baffle with no residual shunt, no significant valvular regurgitation, and a reduction in estimated PASP. Bovine pericardium is a widely used biological material for intracardiac defect correction due to its favorable handling characteristics, low thrombogenicity, and resistance to infection [12]. While these findings demonstrate satisfactory short-term performance of the bovine pericardial baffle, longer-term follow-up is required to confirm its durability.

Finally, DSC was a critical component of our perioperative management strategy. DSC is a well-established surgical strategy for managing severe complications following adult and pediatric cardiac surgery, employed in approximately 1–4% of patients undergoing open-heart surgery [13]. It prevents cardiac and pulmonary compression in cases of significant myocardial or pulmonary edema, thereby improving hemodynamic and respiratory stability [13]. In our patient, severe myocardial edema and hemodynamic instability after multiple closure attempts necessitated leaving the sternum open, with successful closure on postoperative day 4. Recent evidence suggests that DSC is a safe management strategy for high-risk patients in complex cardiovascular surgeries, with no significant increases in mortality or infection rates compared to primary closure. Importantly, a brief duration of DSC (≤4 days) in adults with congenital heart disease is associated with low morbidity and mortality [13,14].

This case has several limitations. As a single case report, our findings may not be generalizable to all patients with single atrium. The intraoperative discovery of aortic dissection was unexpected and added complexity to the procedure, which may not be applicable to other similar cases. We acknowledge that right-heart catheterization was not performed; the elevated estimated PASP likely reflected increased pulmonary flow due to the large intracardiac shunt. Nonetheless, our experience demonstrates that with comprehensive preoperative evaluation, multidisciplinary collaboration, and flexible surgical planning, good outcomes can be achieved even in the most complex scenarios. We recommend that women of childbearing age with undiagnosed or unrepaired congenital heart disease undergo timely echocardiographic screening to avoid pregnancy-related complications, and that high-risk patients be managed in specialized centers with expertise in both adult congenital heart disease and high-risk obstetrics.

## 4. Conclusions

The patient’s survival to age 33 with an unrepaired single atrium suggests long-standing hemodynamic compensation. The cumulative physiological volume overload from repeated pregnancies likely exceeded her cardiovascular reserve, unmasking the underlying defect and leading to diagnosis. Interestingly, the patient also had congenital retinal atrophy with poor vision since childhood, which may have limited her physical activity and, speculatively, contributed to the delayed clinical presentation, adding a thought-provoking perspective to the natural history of this anomaly.

Partial resolution of pregnancy-related hemodynamic load at 6 months postpartum was insufficient to reverse right ventricular enlargement or alleviate symptoms, prompting surgical intervention. The absence of aortic dissection on preoperative imaging, followed by its intraoperative detection after aortic cannulation and CPB, raise the possibility of an iatrogenic mechanism. DSC was successfully performed for severe myocardial edema and circulatory compromise.

The patient showed sustained functional and echocardiographic improvement at 6-month follow-up. While these early results are encouraging, continued follow-up is necessary to confirm long-term durability of the repair and stability of right heart function.

**The patient shared her perspective on the episode of care.**

“Before my surgery, I could barely take care of my newborn baby. Climbing stairs left me breathless, and I worried every day that I might not be there to watch my children grow up. When the doctors told me about the diagnosis during my pregnancy, I was terrified, but the medical team explained everything clearly and gave me hope. After the surgery and the long recovery in the ICU, I finally felt my strength returning. Now, 6 months after the operation, I can carry my baby, walk without shortness of breath, and live a normal life again. I am grateful to the surgical team for giving me a second chance to be a mother to my children.”

## Figures and Tables

**Table 1:** Timeline of diagnosis, treatment, and follow-up.

Date	Event
29 weeks + 6 days of gestation	The patient developed progressive dyspnea and palpitations.
30 weeks + 1 day of gestation	The patient presented to the hospital. TTE revealed a single atrium and estimated PASP of 47 mmHg. A multidisciplinary team recommended oxygen therapy, pulmonary vasodilators, and early termination of pregnancy.
30 weeks + 3 days of gestation	Cesarean section performed with delivery of a healthy neonate.
6 months postpartum	Readmitted for surgical evaluation; estimated PASP of 38 mmHg, right ventricular basal diameter of 48 mm; CTA revealed the diagnosis of single atrium.
Surgery day	Single atrium repair with bovine pericardial patch; intraoperative aortic dissection identified and repaired; decision made to leave sternum open due to myocardial edema.
POD 4	DSC completed.
POD 8	Extubated.
POD 13	Transferred from ICU to general ward.
POD 27	Discharged home.
3-month follow-up	NYHA II; estimated PASP 43 mmHg; right ventricula basal diameter 40.5 mm; LVEF 50.5%; no residual shunt.
6-month follow-up	NYHA I; estimated PASP 40 mmHg; right ventricula basal diameter 40.5 mm; LVEF 50.2%; no residual shunt.

## Data Availability

All relevant data supporting the findings of this case report are included within the article. Further inquiries can be directed to the corresponding author.

## Supplementary Materials

The following supporting information can be downloaded at https://www.mdpi.com/article/10.3390/jcm15176732/s1, Figure S1: Preoperative CT angiography showing mosaic attenuation pattern in the left lung (red rectangular box), suggestive of regional ventilation–perfusion mismatch; Figure S2: Preoperative CT angiography three-dimensional reconstruction demonstrating normal aortic anatomy (aortic sinus approximately 25 mm, ascending aorta approximately 32 mm, aortic arch approximately 22 mm, and proximal descending aorta approximately 18 mm) with no evidence of dissection, aneurysm, or coarctation.
