# Supplementary material for: Single Atrium Repair with Delayed Sternal Closure in a Postpartum Multiparous Woman: A Case Report
Source: J Clin Med. 2026 Aug 30;15(17):6732. doi: 10.3390/jcm15176732 (PMC13565875; doi:10.3390/jcm15176732)
Supplement: Supplementary file 1 [file jcm-15-06732-s001.zip › jcm-4495565-supplementary.pdf]

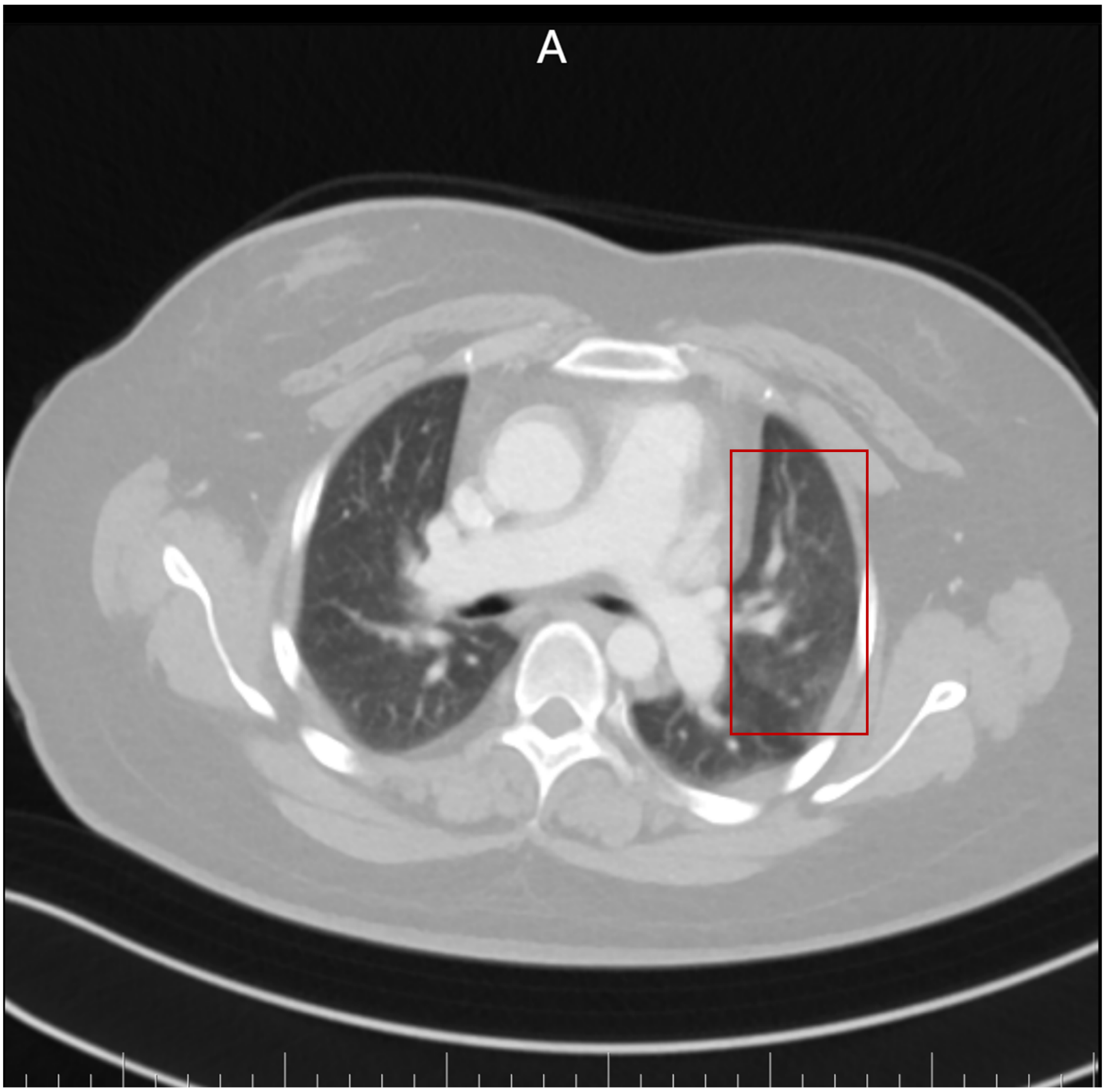

Supplementary Figure S1: Preoperative CT angiography showing mosaic attenuation pattern in the left lung(red rectangular box), suggestive of regional ventilation-perfusion mismatch.

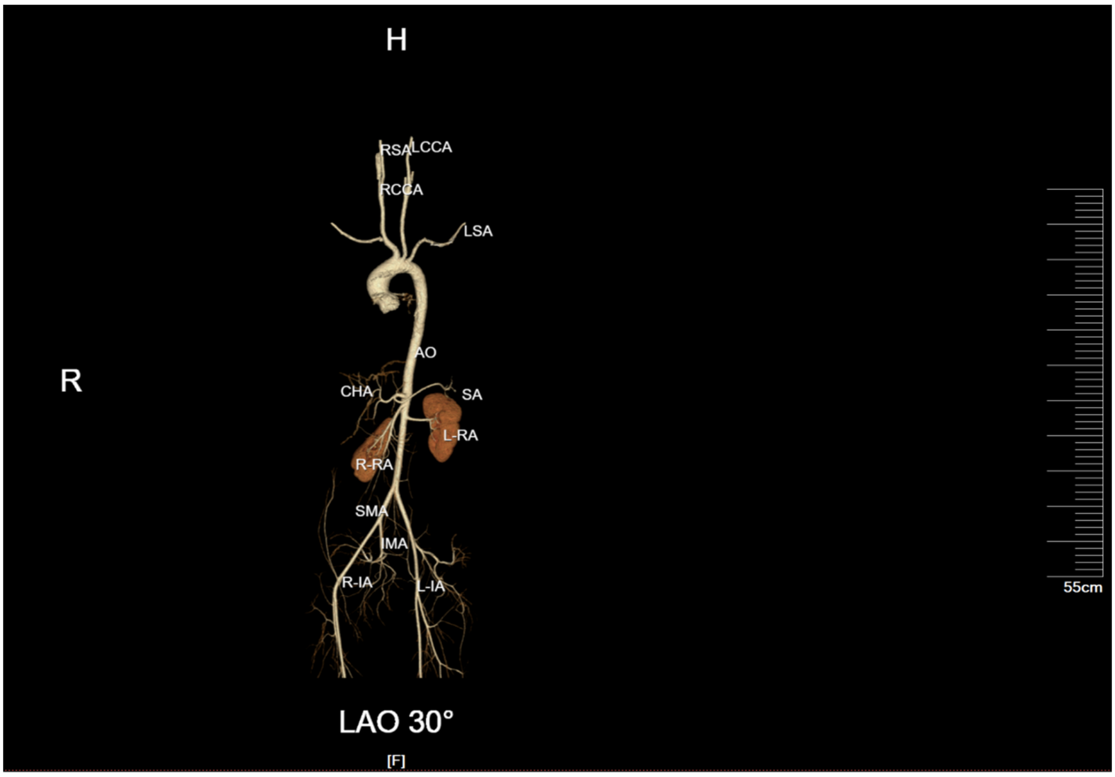

Supplementary Figure S2: Preoperative CT angiography three-dimensional reconstruction demonstrating normal aortic anatomy (aortic sinus approximately 25 mm, ascending aorta approximately 32 mm, aortic arch approximately 22 mm, and proximal descending aorta approximately 18 mm) with no evidence of dissection, aneurysm, or coarctation.
